# HPLC–UV–ESI MS/MS identification of the color constituents of sawwort (***Serratula tinctoria*** L.)

**DOI:** 10.1007/s00216-013-7589-3

**Published:** 2014-01-18

**Authors:** Katarzyna Lech, Katarzyna Witkoś, Maciej Jarosz

**Affiliations:** Chair of Analytical Chemistry, Faculty of Chemistry, Warsaw University of Technology, Noakowskiego 3, 00-664 Warsaw, Poland

**Keywords:** Sawwort, Yellow natural dyestuff, Luteolin glucuronides

## Abstract

Extracts from wool dyed with sawwort (*Serratula tinctoria* L.) obtained with methanol/formic acid and methanol/hydrochloric acid solutions were examined by high-performance liquid chromatography with UV detection coupled with electrospray ionization tandem mass spectrometry. Chromatograms and mass spectra were registered in the negative ion mode under various orifice voltages and collision energies, which enabled us to observe signals corresponding to [M − H]^−^ ions and also Y^−^ and/or Y^−•^ ions, which were further subjected to fragmentation. The results obtained allowed us to define previously unknown constituents of sawwort, which are proposed as specific markers for its identification: chlorogenic acid and its isomers, luteolin-*O*-glucuronides, eriodictyol-*O*-glucuronides, and diosmetin-*O*-glucuronides. Moreover, it was found that during extraction, flavonoid *O*-glucuronides react with methanol in the presence of hydrochloric acid, forming stable O-methylated derivatives.

**Electronic supplementary material:**

The online version of this article (doi:10.1007/s00216-013-7589-3) contains supplementary material, which is available to authorized users.

## Introduction

Sawwort (*Serratula tinctoria* L.) is a source of native European yellow dyestuff that has been used for centuries [1]. 3-*O*-Methylquercetin, apigenin, quercetin, kaempferol [1], and luteolin glycosides [2] are color compounds recommended as specific markers for its identification. However, sawwort has never been identified in archaeological samples [1], despite there being a lot of historical information on its use. This has created doubt concerning the justification for the selection of the above-mentioned markers.

The standard extraction of colorants from textiles involves heating the textiles in a methanolic solution of hydrochloric acid, but as a result, only aglycones can be obtained (strong hydrochloric acid causes hydrolysis of glycosidic bonds). The use of solutions of weak acids, e.g., hydrofluoric, formic, oxalic, or ethylenediaminetetraacetic acid [3, 4], as well as citric or trifluoroacetic acid [5], facilitates release of colorants and preserves glycosidic linkages. To reduce the amount of sample necessary for the analysis, sequential extraction has also been proposed [6].

High-performance liquid chromatography (HPLC) with UV detection coupled with electrospray ionization tandem mass spectrometry (MS/MS) has proven to be a useful tool in analysis of works of art, especially in identification of natural colorants. Fragmentation of *O*-glycosides causes mainly loss of a sugar moiety, whereas in the case of flavonoid aglycones, cleavage of bonds in the central ring and the formation of fragment ions containing ring A or ring B are usually observed. This characteristic fragmentation as well as elimination of CH_3_• (15 Da) from methoxylated flavonoid derivatives and the formation of stable radical anions form the basis for their identification [7].

In this study, HPLC with UV detection coupled with electrospray ionization MS/MS was used to identify components of the extracts obtained from wool dyed with sawwort which could serve as markers, allowing its presence to be proven in samples of historical value. Extraction from the fibers was performed using methanolic solutions of formic or hydrochloric acid. It was found that the additives (hydrolyzing agents) play an important role in ionization and fragmentation of the compounds examined. The data obtained allowed us to propose colorants never reported previously (to the best of our knowledge) and which are essential for identification of sawwort.

## Materials and methods

### Apparatus

Separation and identification of the colorants were conducted using an 1100 LC/MSD system, with spectrophotometric detection, 1200 variable-wavelength detector, and MS/MS detection, 6460 triple-quadrupole liquid chromatography/mass spectrometry (LC/MS) system (Agilent Technologies, Santa Clara, CA, USA). The samples were injected onto a Zorbax SB-Phenyl column (4.6 mm × 150 mm, 3.5 μm, Agilent Technologies) protected by Zorbax SB-Phenyl (4.6 mm × 12.5 mm, 5.0 μm, Agilent Technologies), using a model 7225i injection valve (Rheodyne, Cotati, CA, USA), with a 20-μL loop. The flow rate was 0.5 mL · min^−1^, and elution was performed using 0.15 % (v/v) formic acid in water (solvent A) and methanol (solvent B) with the following program: 0 min, 40 % solvent B; 15 min, 60 % solvent B; 20 min, 70 % solvent B; 27 min, 100 % solvent B; 30 min, 100 % solvent B. The detection wavelength was set at 280 nm. All mass-spectrometric data were recorded in scan mode (*m*/*z* 100–800) or product ion mode (*m*/*z* 50–500, 50–330) of the negative ionization. The capillary voltage was 3.0 kV and the orifice voltage was 100 and 240 V. The nebulizer pressure, nitrogen flow rate, drying gas temperature, drying gas flow rate, and sheath gas temperature were 45 psi, 6 L · min^−1^, 300 °C, 12 L · min^−1^, and 380 °C, respectively, the flow nozzle voltage was 500 V, and the collision energy was – 25 or 35 V. Extraction of the colorants from the fibers was performed with the use of a model 1210 ultrasonic bath (Branson, Danbury, CT USA) as well as with a WB 10 water bath (Memmert, Schwabach, Germany). Raw sawwort extract was centrifuged with use of an MPW-350R laboratory centrifuge (MPW Med. Instruments, Warsaw, Poland).

UV–vis spectra were registered in the range from 240 to 600 nm with the use of a 1100 spectrophotometric diode-array detector (Hewlett-Packard, Waldbronn, Germany). The scan speed and the slit width were 120 nm min^–1^ and 1 nm, respectively.

## Chemicals and materials

Sawwort was donated by the management of the Center for Biological Diversity Conservation of the Polish Academy of Sciences Botanical Garden in Powsin (Poland). Sheep wool came from a rural farm in Kuczbork commune of northern Mazovia (Poland). AlK(SO_4_)_2_∙12H_2_O was from POCH (Gliwice, Poland).

Methanol of LC/MS purity and ethanol of HPLC purity were purchased from POCH (Gliwice, Poland), formic acid of LC/MS purity was purchased from Fisher Scientific (Fair Lawn, NJ, USA), hydrochloric acid (35-38 %) of analytical grade was purchased from AppliChem (Darmstadt, Germany), and demineralized water was obtained from a Milli-Q Elix 3 system (Millipore, Molsheim, France). Luteolin and chlorogenic acid were purchased from Fluka (Buchs, Switzerland), and diosmetin was purchased from ChromaDex (Santa Ana, CA, USA).

### Sample preparation

Preparation of standard solutions, mordanting, and dyeing of wool with sawwort were performed according to [6], as were extractions with methanol(ethanol)/formic acid or methanol(ethanol)/hydrochloric acid. In a two-step extraction, the solution obtained with use of alcohol/formic acid was treated with alcohol/hydrochloric acid. In this case, the times for extraction in the ultrasonic bath and heating in the water bath were twice as short as those in the one-step extraction, 2.5 min and 10 min, respectively.

Raw sawwort extract was prepared by maceration of 20 mg of dry (previously lyophilized) plant material (leaves and stalks) with 5 mL of methanol. It was kept in an ultrasonic bath for 15 min and then in a water bath (at 60 °C) for the next 15 min. The solution obtained was centrifuged for 15 min at 10,000 rpm and filtered through a 0.45-μm poly(ethylene terephthalate) syringe filter. The first five drops were discarded, and only the remaining part of the filtrate after dilution was used for the analysis.

## Results and discussion

Mass spectra of the flavonoid glycosides and aglycone standards examined were registered in the scanning mode at 100 V (orifice voltage) in order to identify deprotonated quasi-molecular ions ([M − H]^−^). Y^−^ and/or Y^−•^ fragment ions were obtained by loss of sugar moieties from glycosides at 240 V, and they were further fragmented by collision-induced dissociation (collision energy 25 or 35 V) and analyzed in the product ion mode.

**Fig. 1:**
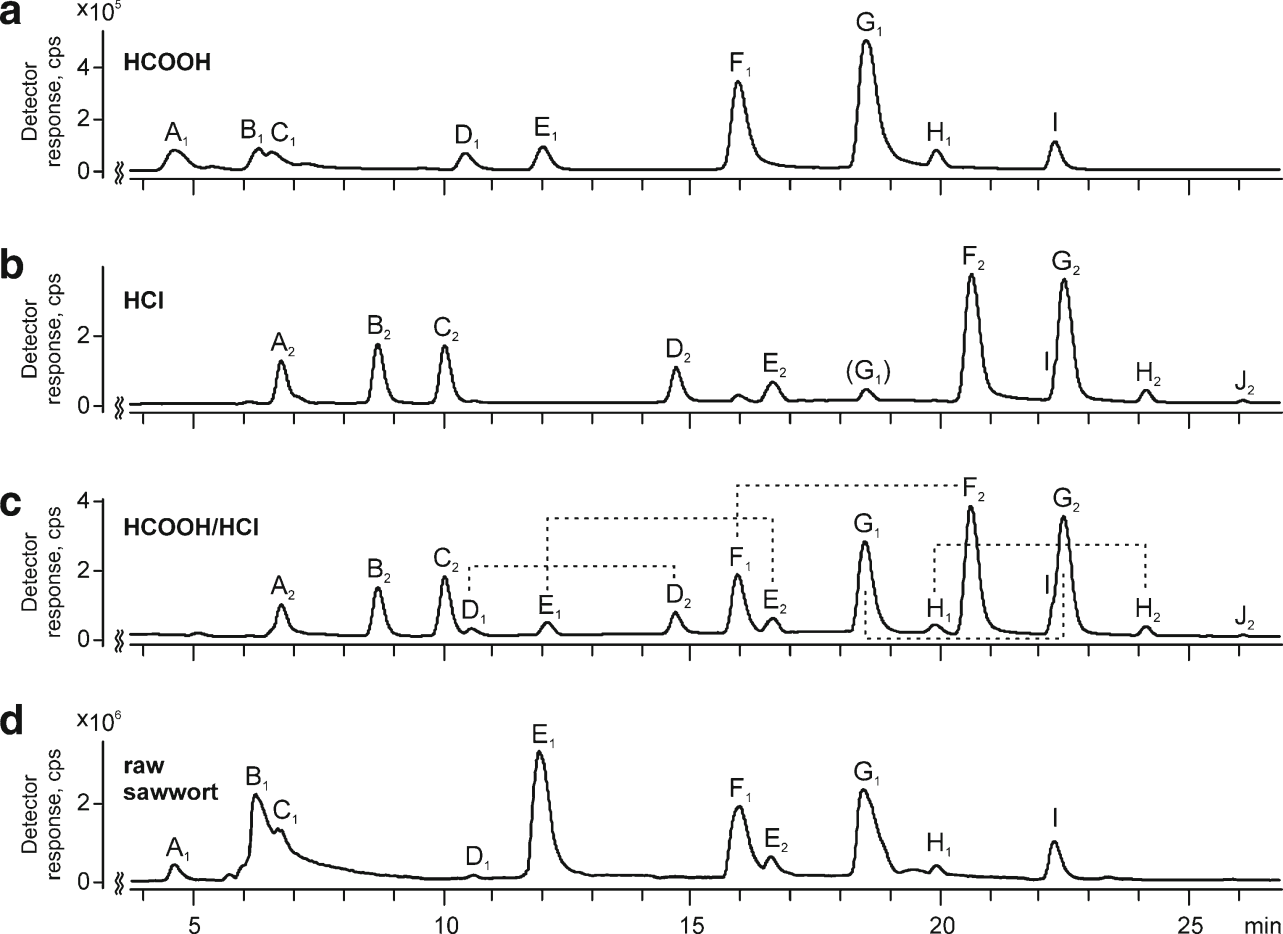
Chromatograms (reconstructed for the [M − H]^−^ ions; see Table 1) of extracts from wool dyed with sawwort obtained using methanolic solutions of *a* formic acid, *b* hydrochloric acid, and *c* formic acid and in a second step modified by addition of hydrochloric acid (mixed extract), and the chromatogram of *d* methanol extract from raw sawwort. Detection by electrospray ionization mass spectrometry was performed in the full scan mode at an orifice voltage of 100 V (for identification details, see Table 1)

In the chromatogram of the methanol/formic acid extract (Fig. 1, chromatogram a), nine peaks were observed, but only two of them, corresponding to chlorogenic acid (C_1_) and luteolin (compound I), could be attributed to available standards. The same nine peaks were also present in the chromatogram of the raw extract (Fig. 1, chromatogram d).

Five of the registered signals, from compounds eluted between 10.6 and 19.9 min (D_1_–H_1_), corresponded to flavonoid *O*-glycosides, and the differences in the *m*/*z* values of their [M − H]^−^ and Y_0_^−^ ions, 176 Da, unambiguously excluded glucose or other hexoses as their structural components. The molecular mass of the neutral loss allowed us to find that glucuronic acid was the sugar part of these flavonoids.

Three of the five compounds discussed (D_1_, F_1_, G_1_) had the same *m*/*z* value as the [M − H]^−^ ions, and their fragmentation paths were identical. The MS/MS spectra of their Y_0_^−^ ions (*m*/*z* 285) corresponded perfectly to the mass spectrum of the luteolin standard (Table 1).

**Table 1:** Characteristics of compounds present in methanolic extracts from wool dyed with sawwort obtained by high-performance liquid chromatography with UV detection coupled with electrospray ionization tandem mass spectrometry (scan and product ion modes)

Type of colorants		Scan, *m*/*z*		Parent ion, *m*/*z*	Product ions, *m*/*z*							*t*_R_ (min)	Peak	Proposed identification
		[M − H]^−^	Y_0_^−^		[R − H]^−^	−R′	−H_2_O	−H_2_O − CO	−H_2_O − CH_2_O	Other				
Isomers of chlorogenic acid	353		353 →	**191**				**135** ^d^			93, 85	4.7	A_1_	Isomer of chlorogenic acid
	353		353 →	**191**				**135** ^d^			93, 85	6.3	B_1_	Isomer of chlorogenic acid
	367		367 →				**161** ^c^		**133** ^c^		85	6.8	A_2_	Isomer of chlorogenic acid methyl ester
	353		353 →	**191**				135^d^			93, 85	7.6	C_1_	Chlorogenic acid
	367		367 →				**161** ^c^	**135** ^c^	**133** ^c^		85	8.7	B_2_	Isomer of chlorogenic acid methyl ester
	367		367 →			**179**	161^c^	**135** ^c^	133^c^			10.0	C_2_	Chlorogenic acid methyl ester
				−CH_3_•	−CO	−2CO		^0,4^A^−^	^1,3^A^−^	^1,3^B^−^	Other			
Flavonoid glucuronides	461	285	285 →				**199**	107	**151**	**133**	175, 83, **65**	10.6	D_1_	Luteolin*-O*-glucuronide
	463	287	287 →					**107**	151	**135**	**65**	12.1	E_1_	Eriodictyol*-O*-glucuronide
	475	299	299 →	**284**	**256** ^a^	**227** ^b^	**199** ^b^		151	133		14.7	D_2_	*O*-Methylluteolin*-O*-glucuronide
	461	285	285 →				199	109	151	**133**	175, 83, 63	15.9	F_1_	Luteolin*-O*-glucuronide
	477	301	301 →	**286**				107	**151**	**135**		16.6	E_2_	*O*-Methyleriodictyol*-O*-glucuronide
	461	285	285 →				199	107	151	**133**	175, 65	18.5	G_1_	Luteolin*-O*-glucuronide
	475	299	299 →	**284**		**227** ^b^	**199** ^b^, 183	**107**	**151**	133	**63**	19.9	H_1_	*O*-Methylluteolin*-O*-glucuronide
	475	299	299 →	**284**	**256** ^a^	227^b^	199^b^, 183	**107**	**151**	133		20.6	F_2_	*O*-Methylluteolin*-O*-glucuronide
	475	285	285 →				199	107	151	**133**	175	22.5	G_2_	Luteolin*-O*-methylglucuronide
	489	299	299 →	**284**	256^a^	**227** ^b^	**199** ^b^	**107**	**151**	133		24.1	H_2_	*O*-Methylluteolin*-O*-methylglucuronide
	285		285 →				199	107	151	**133**	174, 83, 65	22.2	I	Luteolin
	299		299 →	**284**	**256** ^a^	**227** ^b^	199^b^, 183	**107**	**151**	133	83, 65, 63	26.1	J_2_	Diosmetin

*R* is quinic acid (C_7_H_10_O_5_) and R′ is the methyl ester of quinic acid (C_8_H_12_O_5_). Values of *m*/*z* in *bold* correspond to ions with a relative intensity greater than 30 %; values of *m*/*z* in *bold and underlined* correspond to ions with a relative intensity greater than 80 %; *A*_*1*_–*H*_*1*_ are compounds observed only in the methanol/formic acid extract; *A*_*2*_–*H*_*2*_ and *J*_*2*_ are compounds observed only in the methanol/hydrochloric acid extract.

^a^After loss of CH_**3**_• (15 Da)

^b^After loss of CH_4_ (16 Da)

^c^After loss of R′ (188 Da)

^d^After loss of a quinic acid moiety (174 Da)

A similar fragmentation path was observed for the aglycone of another colorant from this group (E_1_), but *m*/*z* 287 of its Y_0_^−^ ion differed from that corresponding to luteolin. However, cleavage of its central (C) ring resulted in the formation of ions at *m*/*z* 107 and 151, the same as in the case of luteolin, corresponding to ^0,4^A^−^ and ^1,3^A^−^, respectively. A difference was observed in *m*/*z* of the ^1,3^B^−^ ions: 135 for colorant E_1_, and 133 for luteolin. This indicated that compound examined reflected the structure of luteolin, but belonged to the flavanone class (with a saturated bond between second and third carbon atoms of the C ring). On the basis of this information, we identified it as eriodictyol.

For compound H_1_ the *m*/*z* values of the [M − H]^−^ and Y_0_^−^ ions were 475 and 299. The similarity of the MS/MS spectrum of the latter to the spectrum of diosmetin (4′-*O*-methylluteolin) allowed us to propose that H_1_ was *O*-methylluteolin-*O*-glucuronide. This suggestion was confirmed by the presence of the intense ion at *m*/*z* 284, formed by the loss of a CH_3_• group (15 Da), which was not observed in MS/MS spectrum of luteolin.

The chromatogram of the sawwort extract obtained with use of methanol/hydrochloric acid solution (Fig. 1, chromatogram b) was similar to the one discussed above. Here, the same number of peaks appeared (A_2_–J_2_), but they were registered at different retention times. Careful interpretation of the full mass spectra allowed us realize that in the case of *O*-glucuronides, hydrochloric acid did not cause hydrolysis of glycosidic bonds (as happens in the case of *O*-hexosides). Moreover, the *m*/*z* values of their [M − H]^−^ ions were 15 Da higher than for analogous quasi-molecular ions of compounds extracted with methanol/formic acid solution. Such results allowed us to hypothesize that during methanol/hydrochloric acid extraction, flavonoid *O*-glucuronides undergo methylation with methanol. To confirm such unusual behavior, two-stage extraction was performed. The solution obtained by extraction performed with the use of methanol/formic acid solution was treated in the second step with hydrochloric acid. In the chromatogram of the new extract (Fig. 1, chromatogram c), all of the previously observed peaks appeared, which clearly indicates the correctness of the above conclusion.

Careful interpretation of the mass spectra allowed us to state that methylation proceeded in two ways. In the case of three compounds (D, E, and F), aglycones were alkylated with methanol, whereas two others (G and H) were glycoside residues. This was confirmed by the data obtained. In the mass spectra of D_2_, E_2_, and F_2_, two main ions were registered: the ions formed by the loss of a fragment of 176 Da corresponding to glucuronic moiety and the ions formed by the loss of a methyl radical from the methoxy group of the aglycone. G_2_ and H_2_ were decomposed by the loss of a glycosidic fragment of 190 Da (larger by 14 Da) corresponding to alkylated glucuronic acid.

**Fig. 2:**
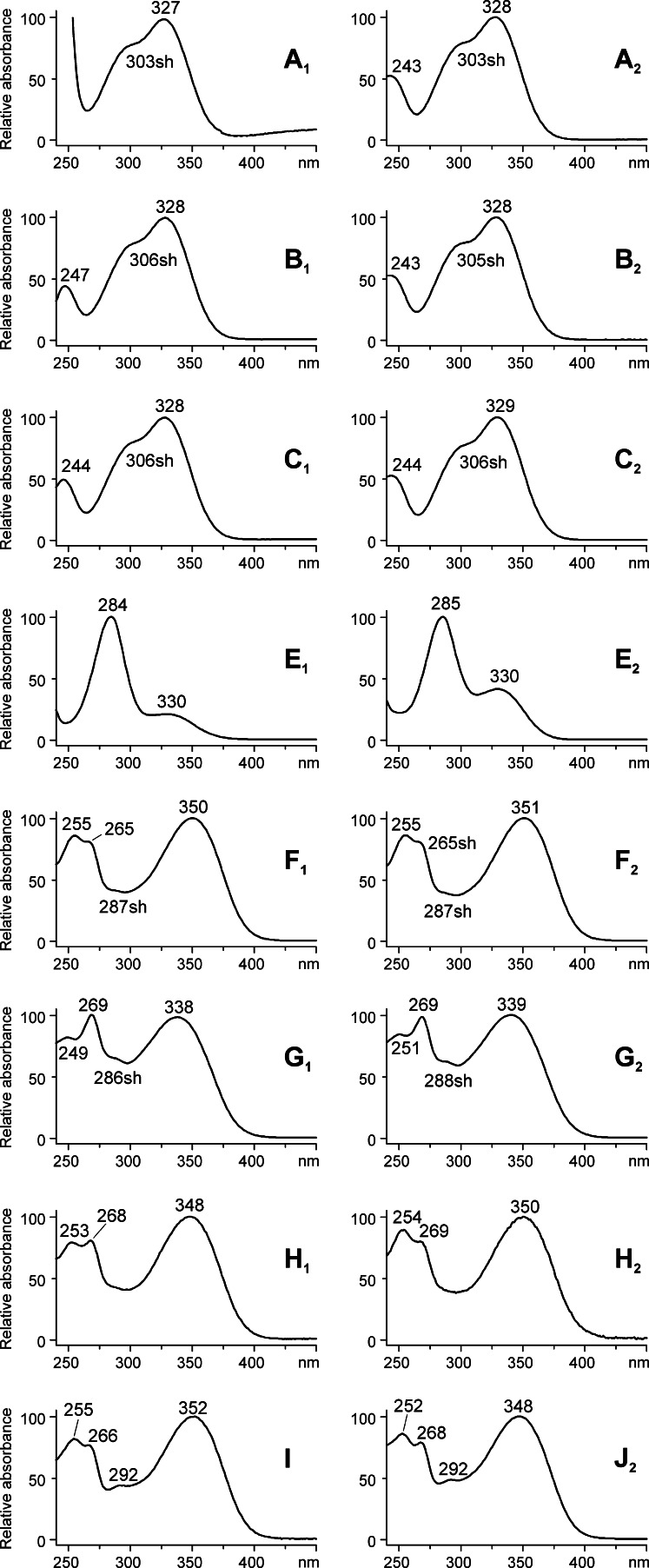
UV–vis (diode-array detection) spectra of compounds registered in chromatograms of extracts from wool dyed with sawwort obtained with the use of a methanolic solution of formic acid (*left column*) and hydrochloric acid (*right column*)

Identification of the compounds based on MS/MS data was also confirmed by UV–vis investigation (Fig. 2). The spectra of luteolin and diosmetin solutions in methanol are typical for flavones, and major absorption maxima are observed in the range from 240 to 400 nm. They are commonly referred to bands related to absorption involving an A-ring benzoyl system (usually 240–280 nm) and a B-ring cinnamoyl system (300–380 nm) [8]. In the case of luteolin (compound I), they are observed at 255, 266, and 352 nm, respectively. Methylation of the hydroxyl group at the 5 or 4′ position leads to a hypsochromic shift of the maximum at 352 nm [8], and in the spectrum of diosmetin (J_2_) it appears at 348 nm.

In the chromatogram of the methanol/formic acid extract, another group of markers specific for sawwort were observed. The three compounds eluted between 4.7 and 7.5 min (A_1_, B_1_, C_1_) were isomers of chlorogenic acid (compound C_1_), as was confirmed by analysis of their MS/MS spectra, and by comparison with the standard. Methylated derivatives of these isomers were also registered in the chromatogram of the methanol/hydrochloric acid extract, but at retention times between 7.5 and 10.0 min. Careful evaluation of the MS/MS spectra allowed us to find that they were probably formed by esterification of the carboxyl group of the quinic acid moiety (Fig. S1).

The experiments were repeated using ethanolic solutions, and in the extracts obtained ethylated derivatives of flavonoid glucuronides were found. This created a basis for the conclusion that the alkylation reaction was not specific and not limited to methanol. Methanol and ethanol are stronger bases than phenol or flavonoid aglycones, and are much stronger bases than glucuronic acid [9]. Alcohol molecules can be reversibly protonated by strong acids, such as hydrochloric acid, to yield oxonium ions (CH_3_OH_2_^+^), allowing nucleophilic attack by a flavonoid with simultaneous loss of water (Fig. S2). Transfer of a proton from the new oxonium ion to a water molecule regenerates the acid catalyst and gives an O-alkylated product. Thus, alkylation of flavonoid glucuronides, and not their hydrolysis, occurs undoubtedly owing to the stronger bond between the aglycone and the glucuronide moiety than between the aglycone and the glucoside.

## Conclusion

In the study performed, sawwort was investigated and carefully characterized. Its colorants, not previously reported to the best of our knowledge, and not found in other yellow dyes of apparently similar composition, were identified as the main components. The following compounds are proposed as specific markers for the natural dyestuff examined: chlorogenic acid and its isomers, various luteolin-*O*-glucuronides, and, present in a smaller amount, eriodictyol-*O*-glucuronides and diosmetin-*O*-glucuronides. Moreover, it was found that hydrochloric acid present in the extractant causes alkylation instead of hydrolysis of glycosidic colorants of sawwort. The results presented create the basis for univocal distinction of sawwort from other natural yellow dyestuffs.

## Electronic supplementary material

Below is the link to the electronic supplementary material.

ESM 1 (PDF 349 KB)
