# Supplementary material for: HPLC–UV–ESI MS/MS identification of the color constituents of sawwort (Serratula tinctoria L.)
Source: Anal Bioanal Chem. 2014 Jan 18;406(15):3703–8. doi: 10.1007/s00216-013-7589-3 (PMC4026629; doi:10.1007/s00216-013-7589-3)
Supplement: Supplementary file 1 — (PDF 349 KB) [file 216_2013_7589_MOESM1_ESM.pdf]

Analytical and Bioanalytical Chemistry

Electronic Supplementary Material

HPLC–UV–ESI MS/MS identification of the color constituents of sawwort (*Serratula tinctoria* L.)

Katarzyna Lech, Katarzyna Witkoś, Maciej Jarosz

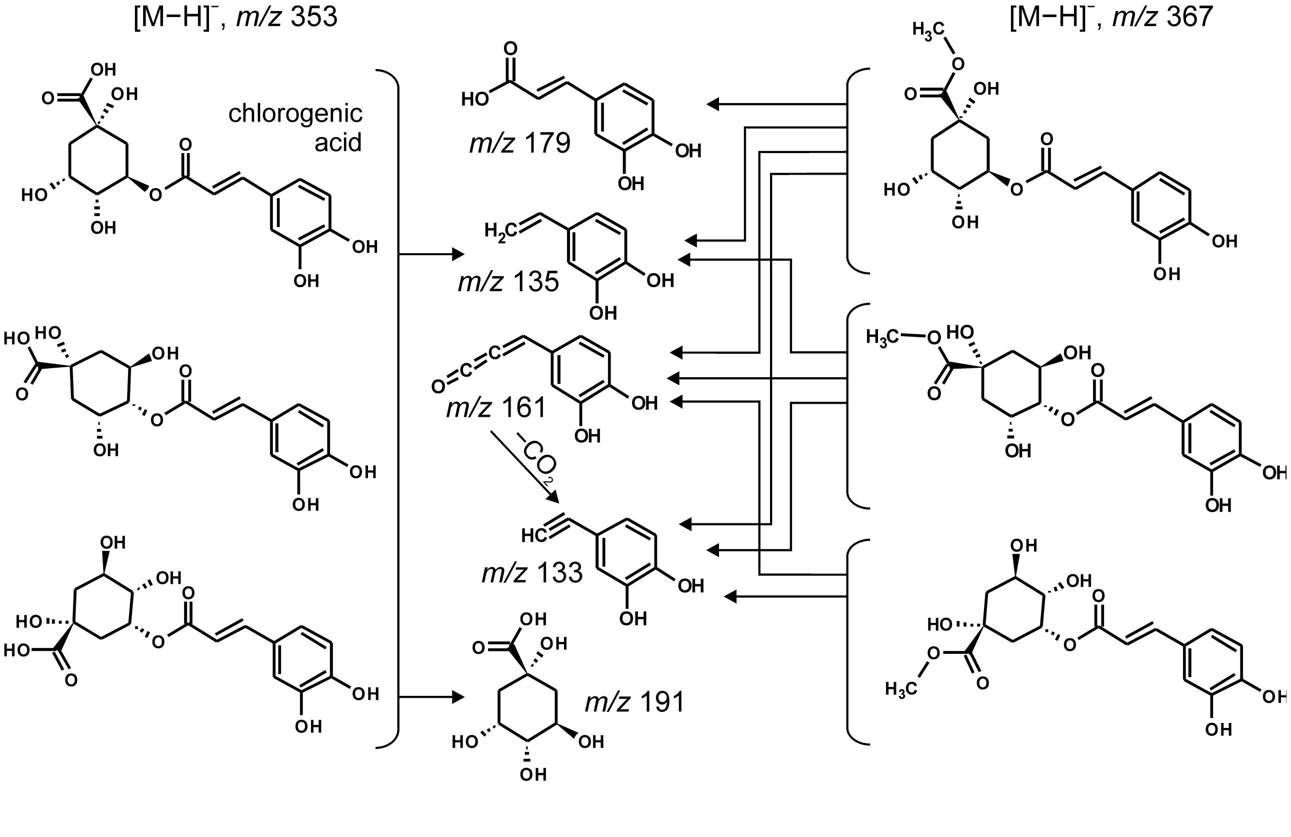

Fig. S1. Proposed structures and fragmentation pathways of chlorogenic acid isomers (left) and their methylated analogue (right)

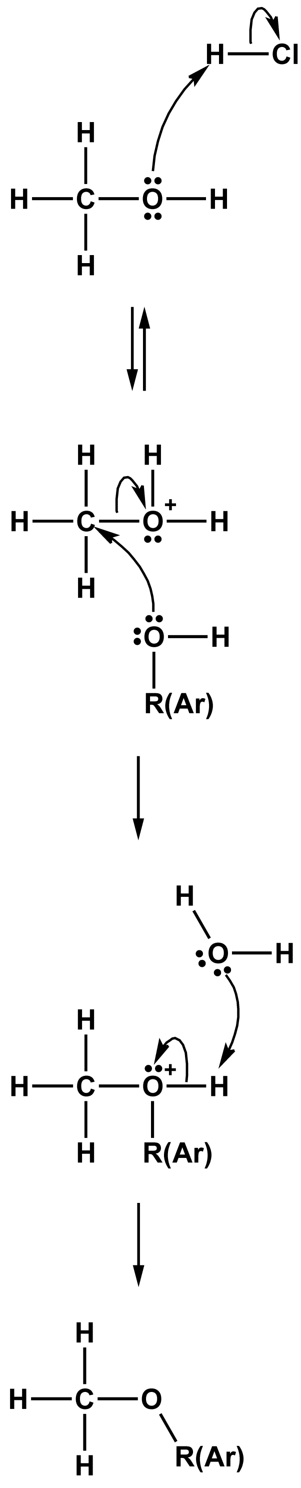

Fig. S2. Mechanism of an acid-catalyzed methylation of flavonoid glucuronides (R(Ar))
